# Oliver Wendell Holmes

**Published:** 1891-01

**Authors:** 


### Oliver Wendell Holmes "De Senectute."

The present age is remarkable, among other things, for the physical and intellectual vitality of its old men. It is not only that—thanks to the advance of medicine, preventive and curative—we have more old men among us than formerly, but that, so far from lagging superfluous on the stage of life, the veterans continue to play most of the principal parts with all the ardor and sometimes more than the versatility, of youth. In all departments of modern life—in politics, in the church, in the army, in science, and in literature, we see men whose years are far beyond the psalmist's limit predominate not only in influence and reputation, but in the amount and quality of their actual work. Among the octogenarians on whose mental brow times writes no wrinkles, none is more interesting to medical men than the genial "Autocrat" of so many hearts both in England and America, whom, in spite of the poet's days which he wears so gracefully, we are proud to claim as a professional brother; Oliver Wendell Holmes, who celebrated his 81st birthday on August 29, is still one of the most vivacious of men; age can not wither the freshness of his interest in life, nor deaden the cheerful sparkle of his style. Even of "crabbed age" and the inevitable sorrows and bereavements which it brings with it, he writes with an easy wit, quite untinged with cynicism, which *circum praecordia ludit* and brightens the dismal subject so as to make it amusing to his fellow-sufferers. From the purely medical point of view his account of his mode of life is instructive as well as interesting. For a long time back, he says, he has taken extreme care of himself. Never robust, he was still wiry in his earlier and maturer life; but since he reached the age of 80 his hygienic vigilance is unceasing. The rooms which he daily occupies are equipped with barometers, thermometers, ærometers—with every kind of instrument, in short, to prevent his incurring the slightest risk of taking cold. As pneumonia is the deadliest foe of old age, he does his utmost to keep it at a distance. He never gets up during winter until he knows the exact temperature, or takes his bath without having the water accurately tested. He lives by rule, and the rule is inflexible. His time is scrupulously divided—so much allotted to reading, so much to writing, so much to exercise, so much to recreation. His meals are studies of prudence and digestion. He understands the specific qualities of all ordinary foods, and never departs from the severest discretion in eating. To this strict hygienic discipline Dr. Holmes attributes his good health, and the retention of his mental vigor.—*Brit. Med. Journal.*

